# Identification and functional characterization of the *CYP51* gene from the yeast *Xanthophyllomyces dendrorhous* that is involved in ergosterol biosynthesis

**DOI:** 10.1186/s12866-015-0428-2

**Published:** 2015-04-25

**Authors:** Kritsye Leiva, Nicole Werner, Dionisia Sepúlveda, Salvador Barahona, Marcelo Baeza, Víctor Cifuentes, Jennifer Alcaíno

**Affiliations:** Departamento de Ciencias Ecológicas y Centro de Biotecnología, Facultad de Ciencias, Universidad de Chile, Las Palmeras 3425, Casilla 653, Ñuñoa, Santiago, Chile

**Keywords:** Cytochrome P450 enzyme systems (P450), Astaxanthin, Sterols, Sterol C14-demethylase

## Abstract

**Background:**

*Xanthophyllomyces dendrorhous* is a basidiomycetous yeast that synthesizes astaxanthin, a carotenoid with great biotechnological impact. The ergosterol and carotenoid synthetic pathways derive from the mevalonate pathway and involve cytochrome P450 enzymes. Among these enzymes, the CYP51 family, which is involved in ergosterol biosynthesis, is one of the most remarkable that has C14-demethylase activity.

**Results:**

In this study, the *CYP51* gene from *X. dendrorhous* was isolated and its function was analyzed. The gene is composed of ten exons and encodes a predicted 550 amino acid polypeptide that exhibits conserved cytochrome P450 structural characteristics and shares significant identity with the sterol C14-demethylase from other fungi. The functionality of this gene was confirmed by heterologous complementation in *S. cerevisiae*. Furthermore, a *CYP51* gene mutation in *X. dendrorhous* reduced sterol production by approximately 40% and enhanced total carotenoid production by approximately 90% compared to the wild-type strain after 48 and 120 h of culture, respectively. Additionally, the *CYP51* gene mutation in *X. dendrorhous* increased *HMGR* (hydroxy-methylglutaryl-CoA reductase, involved in the mevalonate pathway) and *crtR* (cytochrome P450 reductase) transcript levels, which could be associated with reduced ergosterol production.

**Conclusions:**

These results suggest that the *CYP51* gene identified in *X. dendrorhous* encodes a functional sterol C14-demethylase that is involved in ergosterol biosynthesis.

**Electronic supplementary material:**

The online version of this article (doi:10.1186/s12866-015-0428-2) contains supplementary material, which is available to authorized users.

## Background

*Xanthophyllomyces dendrorhous* is a basidiomycete yeast that has been mostly studied for its ability to synthesize xanthophyll astaxanthin as its main carotenoid. Astaxanthin (3,3’-dihydroxy-β,β-carotene-4-4’-dione) takes third place in the global market for carotenoids, which reached $226 million in the year 2010 and is expected to exceed $250 million by the year 2018 [1]. This carotenoid is currently used in aquaculture for salmon flesh pigmentation and as a supplement for the diets of farm chickens to produce a stronger yolk and flesh pigmentation [2]. Additionally, astaxanthin has strong antioxidant properties and has been proposed to play a protective role against oxidative stress in yeast [3-5]. Recent findings suggested that astaxanthin supplementation could be beneficial as a treatment for several degenerative diseases in addition to other potential benefits for human health [6-9]. To date, *X. dendrorhous* is the only known organism that synthesizes astaxanthin from β-carotene through a cytochrome P450 enzyme system [10-13], suggesting that this yeast has developed a unique P450 system specialized for the production of this carotenoid.

Cytochrome P450 proteins (P450s or CYPs) represent a large superfamily of heme-containing monooxygenases that are distributed throughout three domains of life [14] and play significant roles in the oxidative metabolism of a wide range of exogenous and endogenous substrates [15]. CYP enzymes are involved in the biosynthesis of many physiologically important compounds, such as sterols, steroid hormones, fatty acids and vitamins [16], and a vast array of secondary metabolites in plants, insects and fungi [17]. Additionally, these enzymes are the main catalysts involved in the activation and detoxification of different xenobiotics, such as drugs, pesticides, carcinogens and environmentally contaminating chemicals [18]. CYP enzymes require the reducing equivalents from NADPH or NADH, which are generally transferred to CYPs through a redox partner to catalyze the general reaction RH + O_2_ + 2e^−^ + 2H^+^ → ROH + H_2_O [19].

Only two CYP enzymes have been described in *X. dendrorhous*: the astaxanthin synthase that is involved in astaxanthin biosynthesis and CYP61, which participates in ergosterol biosynthesis [20]. Considering the biological relevance of this protein family and the involvement of a unique system in *X. dendrorhous*, in this work we describe a third CYP in this yeast: CYP51. CYP51 is one of the first modifying enzymes involved in sterol synthesis. It catalyzes the C14 demethylation of lanosterol in yeasts during ergosterol biosynthesis and is considered to be an ancestral CYP from which other CYPs evolved [21].

## Results and discussion

### Cloning and sequence analysis of the *X. dendrorhous CYP51* gene

**Figure 1:**
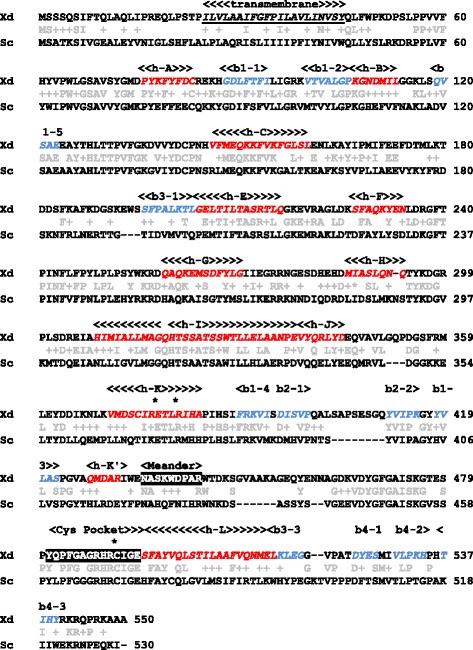
Sequence alignment between *X. dendrorhous* CYP51 and *S. cerevisiae* ERG11 proteins and prediction of structurally conserved motifs in CYP51. Amino acid alignment between the deduced CYP51 sequence from *X. dendrorhous* (Xd) strain UCD 67–385 and the *S. cerevisiae* (Sc) strain S288c ERG11 (lanosterol C14-demethylase) protein [Swiss Protein: P10614]. Amino acid differences with the same properties are denoted with a plus (+). Structural elements are highlighted with the name of the corresponding feature above them: possible transmembrane helix (underlined and italics), alpha helices (red and italics), beta-sheets (blue and italics), meander loop and Cys pocket (white highlighted in black). The asterisks (*) indicate the three totally conserved amino acids among cytochromes P450. Secondary structural elements were predicted with the CYP450 Engineering database [26], and the potential transmembrane region was predicted with TMpred [52].

Using bioinformatic analyses of the genomic and transcriptomic data of *X. dendrorhous* available in our laboratory with the *S. cerevisiae ERG11* gene as a query [GenBank: NM_001179137], we identified a putative *X. dendrorhous CYP51* gene. This gene was uploaded into the Genbank database [GenBank: KP317478]. The primers CYP51 up.F and CYP51dw.R were designed based on this gene sequence and used to amplify genomic DNA from strain UCD 67–385. The obtained PCR-product of approximately 4,300 bp was inserted into the *Eco*RV site of the pBluescript SK- plasmid, generating plasmid pBS-g*CYP51*. Similarly, the cDNA version (ORF: 1,653 bp) of the *CYP51* gene was obtained by RT-PCR from *X. dendrorhous* total RNA using the primer set cCYP51.F + cCYP51.R. This cDNA was ligated into the *Eco*RV site of the pBluescript SK- plasmid to generate plasmid pBS-c*CYP51*. The sequences of the genomic and cDNA versions were determined in both strands and compared, revealing that the *CYP51* gene from *X. dendrorhous* consists of 10 exons of 141, 70, 47, 63, 60, 462, 86, 233, 83 and 408 bp and 9 introns of 356, 95, 79, 80, 86, 110, 90, 87 and 90 bp. The *CYP51* gene was predicted to encode a 550 amino acid protein with an expected molecular weight of 61.8 kDa and pI of 6.64. This protein shares 48% identity and 65% similarity at 91% coverage with the *S. cerevisiae* C14-demethylase [ERG11, Swiss-Prot: P10614] (Figure 1), which belongs to the cytochrome P450 protein family and is involved in the third step of ergosterol biosynthesis from squalene: the conversion of lanosterol to 4,4-dimethylcholesta-8,14,24-trienol [22,23].

Amino acid sequences are highly diverse among the cytochrome P450 protein family; however, their structural fold is highly conserved [24]. In the deduced CYP51 protein from *X. dendrorhous*, several cytochrome P450 secondary structural elements were predicted using the CYP450 Engineering database, including alpha helices A, B, C, E, F, G, H, I, J, K, K’ and L, beta-sheets 1–1,1-2, 1–5, 3–1, 1–4, 2–1, 2–2, 1–3, 3–3, 4–1, 4–2 and 3–2, the meander loop involved in the stabilization of the tertiary structure and heme binding, and the Cys pocket that contains the conserved cysteine involved in heme binding [25] (Figure 1). Identification of P450s across biological kingdoms depends largely on the identification of two P450 signature motifs: ExxR at the K-helix and FxxGxRxCxG (also known as the CxG motif) at the Cys-pocket. These motifs contain three totally conserved amino acids: the glutamic acid and arginine of the ExxR motif and the cysteine in the Cys pocket that serves as a fifth ligand for the heme iron [26]. Site-directed mutagenesis of these three invariant residues in most CYPs results in inactive and misfolded P450 isoforms, suggesting their importance in maintaining the P450 structure [27,28]. All of these motifs were recognized in the *X. dendrorhous* deduced CYP51 protein, including the three invariant P450 residues. A threonine and a leucine were found at the second and third positions of the ExxR motif; these are the preferred residues at these positions in P450s, including the CYP51 family [29]. Moreover, the CxG motif of the *X. dendrorhous* CYP51 protein (FGAGRHRCIG) maintains the preferred residues described for the CYP51 family [29]. This finding is interesting because instead of the conserved arginine at the sixth position of this motif, CYP51 contains a histidine surrounded by arginines. Additionally, a putative hydrophobic trans-membrane segment at the CYP51 amino terminus was predicted. This finding represents an important feature that allows class II P450 enzymes to anchor to the endoplasmic reticulum [30]. These observations strongly suggest that the identified *X. dendrorhous* gene encodes a cytochrome P450 enzyme, specifically CYP51 with lanosterol demethylase activity.

**Figure 2:**
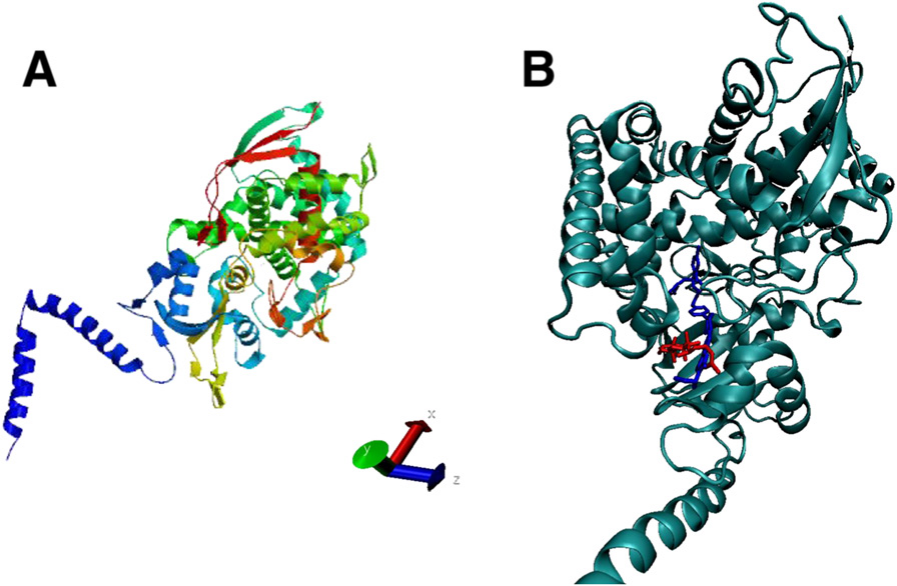
Three-dimensional model and docking of the *X. dendrorhous* CYP51 deduced protein. **(A)** The model was generated by the SwissModel web server [54] using the *S. cerevisiae* ERG11 protein with lanosterol as the template ligand (model code. 4 lxj.1). **(B)** The AutoDock4 program [31] was used to perform automated docking to predict the binding sites of lanosterol (red) and itraconazole (blue) in the obtained model of the CYP51 protein from *X. dendrorhous*.

The deduced *X. dendrorhous* CYP51 protein was modeled (Figure 2A) with the homology modeling technique using the SwissModel web server. The *S. cerevisiae* ERG11 protein [PDB: 4 lxj.1], which shares 48% sequence identity, was used as a template. Additionally, possible protein-ligand binding interactions between the CYP51 protein model and the potential lanosterol and itraconazole ligands were predicted with the protein docking technique using the AutoDock4 program [31] (Figure 2B). According to the results, the azole antifungal fits in a similar way to the substrate lanosterol in a hydrophobic groove and interacts with Met-315 and the P-methyl group of Thr-322. Previous studies have also reported these residues as binding sites of azole antifungals in cytochrome P450 proteins [32]. Therefore, the itraconazole azole binds to the CYP51 protein in the substrate-binding pocket of the enzyme, thus blocking its enzymatic catalysis.

### Functional analysis of the *X. dendrorhous CYP51* gene

The functionality of the *X. dendrorhous CYP51* gene was evaluated by two different approaches: heterologous complementation in *S. cerevisiae* and gene mutation in *X. dendrorhous*.

**Figure 3:**
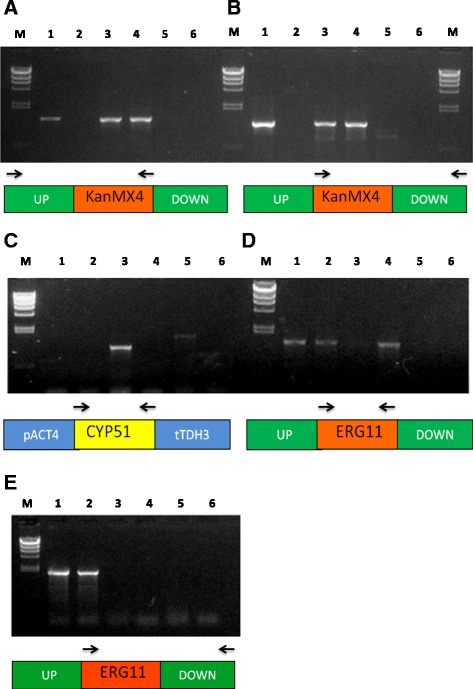
PCR-based analyses of *S. cerevisiae erg11* ^−^ strains carrying the YEpNP-gERG11 and YEpNP-cCYP51 vectors. PCR analyses to confirm the presence of the *kanMX* gene at the *ERG11 locus* (**panel A**: primers CYP51ScExt.F and KanMX4.R2 and **panel B**: primers KanMX4.F2 and CYP51ScExt.R), the presence of the *X. dendrorhous CYP51* gene (**panel C**: primers RTCYP51.F and CYP51.Rb), the presence of the *ERG11* gene (**panel D**: gERG11.F and gERG11.R) and the absence of the *ERG11* gene in the *S. cerevisiae* genome (**panel E**: gERG11.F and CYP51ScExt.R). Template DNA in each lane: *S. cerevisiae* parental diploid strain erg11 +/− (Lane 1), *S. cerevisiae* S288c strain (Lane 2), *S. cerevisiae* Sc-hCYP51 strain (Lane 3), *S. cerevisiae* Sc-hERG11 strain (Lane 4), *X. dendrorhous* UCD 67–385 strain (Lane 5), negative control without DNA (Lane 6). Molecular size marker Lambda/*Hin*d III (Lane M: 23.1, 9.4, 6.6, 4.4, 2.3, 2.0 and 0.6 kb). A schematic diagram is included to represent the primer sets (shown in arrows) that were used. The UP and DOWN regions correspond to regions located 300 bp upstream and downstream of the *S. cerevisiae ERG11* gene, respectively. Region KanMX4 corresponds to the geneticin (G418) resistance module and the pACT4 and tTDH3 regions correspond to the *S. cerevisiae* promoter and terminator region in YEpNP.

The heterologous complementation analyses were performed using a diploid *S. cerevisiae* that is heterologous for the *ERG11* gene (Sc-*erg*11^+/−^ strain), because a null mutant is not viable [33]. The *S. cerevisiae ERG11* gene and the cDNA of the *X. dendrorhous CYP51* gene were ligated into the *S. cerevisiae* expression vector YEpNP, generating plasmids YEpNP-gERG11 and YEpNP-cCYP51; then, these plasmids were used to transform the Sc-*erg*11^+/−^ strain. Several transformants were recovered in each case, and at least three transformants of each type were randomly chosen to confirm that they contained the respective plasmid. Given that all of the analyzed transformants showed a positive result in this analysis, one of each (named Sc-g*ERG11*Sc and Sc-c*CYP51*Xd, respectively) was chosen for sporulation to obtain haploid strains. After 6 days of incubation in sporulation media agar plates, the formation of asci was confirmed by optical microscopy. The asci were broken and seeded onto SD agar plates supplemented with G418 to confirm the presence of the *S. cerevisiae ERG11* mutant allele, and with uracil, histidine, lysine and methionine to sustain all possible auxotrophies in the resulting haploid strains. Although the haploid cells were enriched by diethyl ether treatment [34], it is also possible to obtain diploid strains among the G418-resistant strains. Because of this, haploid strains were first selected according to their auxotrophy for methionine and lysine, both of which are heterozygous markers in the original diploid parental strain. Subsequently, the haploid condition of the *ERG11* mutant allele and its complementation of the expression vector were confirmed by PCR analyses using comprehensive sets of primers (Figure 3). After these analyses, a single strain that fulfilled the requirements for heterologous expression studies carrying the YEpNP-g*ERG11* or the YEpNP-c*CYP51* vector was chosen; these strains were named Sc-h*ERG11* and Sc-h*CYP51*, respectively.

The strains Sc-h*ERG11* and Sc-h*CYP51* did not show significant differences in their growth curves when cultured in YM medium at 22°C with constant agitation (Additional file 1: Figure S1). Additionally, sterols were extracted after 10, 24 and 56 h of cultivation (Table 1). When the sterol compositions were analyzed by RP-HPLC, all strains showed the same sterol pattern, with one main sterol produced with 13 min of retention time. This sterol was confirmed to correspond to ergosterol after co-injecting each sample with standard ergosterol. In general, strain Sc-h*CYP51* showed a lower sterol content than strains Sc-*erg*11^+/−^ and Sc-h*ERG11* at all analyzed time points. However, the fact that strain Sc-h*CYP51* was viable and able to produce ergosterol indicates that the *CYP51* gene from *X. dendrorhous* complements the *erg11* null mutation in *S. cerevisiae.* Therefore, this gene encodes a lanosterol C14-demethylase that is able to couple with the endogenous *S. cerevisiae* cytochrome P450 reductase *in vivo*.

**Table 1:** **Sterols obtained from** ***S. cerevisiae*** **strains used in this study (mg per g of dry yeast)**

	***S. cerevisiae*** **strains**								
	**(Sc-** ***erg*** **11** ^**+/−**^ **)**			**Sc-h** ***ERG11***			**Sc-h** ***CYP51***		
**Cultivation Time (h)**	**10**	**24**	**56**	**10**	**24**	**56**	**10**	**24**	**56**
**Total sterols**	25 ± 5	11 ± 1	21 ± 3	21 ± 8	16 ± 5	38 ± 7	19 ± 3	9 ± 1	20 ± 2
**Ergosterol (11 min)**	23 ± 5	10 ± 1	20 ± 2	17 ± 2	15 ± 4	34 ± 6	16 ± 3	9 ± 3	18 ± 2
**Peak 2 (12.5 min)**	ND	0.03 ± 0.02	0.2 ± 0.3	ND	0.06 ± 0.02	0.4 ± 0.4	ND	ND	0.21 ± 0.04
**Peak 3 (15 min)**	3 ± 1	0.7 ± 0.1	1.45 ± 0.03	4 ± 1	1.5 ± 0.4	3.1 ± 0.4	2.4 ± 0.6	0.7 ± 0.1	1.6 ± 0.1

ND: Not detected. Table values correspond to the average result from three independent cultures ± standard deviations. The RP-HPLC retention time is indicated in parenthesis.

To evaluate the functionality of *CYP51* in *X. dendrorhous*, *cyp51* mutants were created by replacing the *CYP51 locus* with an antibiotic resistance module through homologous recombination as previously described [35]. For this purpose, the wild-type strain CBS 6938 was chosen because it has been shown to be aneuploid, and hemizygous mutants can be obtained after only one transformation event [12]. Before transformation, the *CYP51* gene from strain CBS 6938 was sequenced, uploaded to the Genbank database [Genbank: KP317479] and compared to the sequence from strain UCD 67–385. Minimal differences were found between these two strains, which possessed 99.707% identity in the region from the start to the stop translation codons. After CBS 6938 transformation with linear plasmid pBS-*cyp51*/*hph*, a hygromycin B-resistant transformant was obtained. Although it was confirmed that the *CYP51 locus* was indeed replaced by the resistance module in this strain, the *CYP51* gene could still be amplified using a comprehensive set of primers, suggesting that this strain was heterozygous for the *CYP51 locus*. Therefore, this strain was named CBS*-CYP51*^+/−^. This strain was still a useful model to evaluate the effect of the *CYP51* mutation in *X. dendrorhous;* because the deletion of this gene has been shown to be lethal to the cells of other yeast strains, a *cyp51* null mutant would most likely not be viable [36]. Interestingly, the mutant strain had a more intense reddish color that was discernable to the naked eye than the wild type strain, suggesting that it produces more carotenoids than the parental strain. To confirm this observation and to evaluate phenotypic variations between these two strains, growth curves were constructed and sterols and carotenoids were extracted and quantified after 48 and 120 h of cultivation.

The growth curves of both strains did not show significant differences at the lag and exponential phases of growth; however, strain CBS*-CYP51*^+/−^ reached a lower OD_600_ during the stationary phase of growth (P-value < 0.05, Student's *t* test) (Additional file 1: Figure S1). After 48 h of cultivation, the wild-type strain accumulated approximately 70% more sterols than the mutant strain; however, after 120 h of culture, there were no statistically significant differences between the strains (Table 2). This outcome may be the result of a regulation mechanism that modulates sterol biosynthesis according to the metabolic state of the yeast, which is reduced during the stationary phase of growth. Similarly, previous studies in filamentous fungi and in yeast have reported reduced ergosterol accumulation during the stationary phase of growth [20,37]. Moreover, both strains showed the same sterol composition, with ergosterol representing close to 100% of the identified sterols; this result was confirmed by co-injection of the samples with standard ergosterol in RP-HPLC analysis. An additional two unidentified sterols could be detected in both strains with retention times of 12.5 and 15 min; however, these sterols were found in a significantly lower proportion.

**Table 2:** **Sterols (mg per g of dry yeast) and carotenoids (μg per g of dry yeast) obtained from the** ***X. dendrorhous*** **used in this study**

	***X. dendrorhous*** **strains**			
	**CBS 6938**		**CBS** ***-CYP51*** ^**+/−**^	
**Cultivation Time (h)**	**48**	**120**	**48**	**120**
Sterols:	5.6 ± 0.2	3.8 ± 0.4	3.2 ± 0.7	4.9 ± 0.8
Total Carotenoids:	24 ± 5 (100)	256 ± 18 (100)	325 ± 115 (100)	486 ± 54 (100)
Astaxanthin	19 ± 4 (78)	174 ± 23 (68)	95 ± 27 (29)	203 ± 20 (42)
Phoenicoxanthin	2.5 ± 0.7(10)	30.0 ± 0.8 (12)	37 ± 15 (11)	74 ± 14 (15)
Canthaxanthin	ND	ND	9 ± 5 (3)	12 ± 6 (3)
Echinenone	ND	ND	4 ± 7 (1)	ND
β-carotene	ND	12 ± 6 (5)	67 ± 33 (21)	55 ± 15 (11)
Lycopene	ND	ND	7 ± 6 (2)	ND
Monocyclic carotenoids	2.8 ± 0.8 (11)	40 ± 3 (16)	107 ± 40 (33)	149 ± 27 (31)

ND: Not detected. Table values correspond to the average result from three independent cultures ± standard deviations. Percentage relative to total carotenoids is indicated in parenthesis. Monocyclic carotenoids include: γ-carotene, keto-γ-carotene, hydroxy-keto-γ-carotene, torulene and hydroxy-keto-torulene.

The mutant strain contained a higher amount of carotenoids (P-value < 0.05, Student's *t* test), with an approximately 1.9-fold increase after 120 h of culture. Previous studies on carotenogenesis in *X. dendrorhous* have shown that this process is induced by the end of the exponential and early stationary phases of growth, coinciding with the point at which glucose is completely exhausted [38-40]. Therefore, it is expected that the major differences in carotenoid content can be better appreciated in older cultures. However, the CBS*-CYP51*^+/−^ strain also had a higher carotenoid content after 48 h of culture, suggesting that carotenogenesis starts earlier in this strain. Moreover, carotenoid composition also differed between both strains, with a reduced astaxanthin proportion in the CBS*-CYP51*^+/−^ strain (Table 2). Interestingly, changes in the carotenoid composition showed similarity to cultures of *X. dendrorhous* in which the *crtI* gene (encoding the phytoene desaturase) was over-expressed [41], resulting in an increased monocyclic carotenoid proportion in CBS*-CYP51*^+/−^ cells. Moreover, β-carotene and xanthophyll intermediaries such as phoenicoxanthin and canthaxathin are increased during the synthesis of astaxanthin from β-carotene in CBS*-CYP51*^+/−^, suggesting that the cytochrome P450 system involved in these steps may be weakened. It is likely that this change is the consequence of the reduced *CYP51* gene dose in CBS*-CYP51*^+/−^, which could favor the activity of the cytochrome P450 systems involved in ergosterol biosynthesis at the expense of astaxanthin synthesis. This idea is supported by the higher cytochrome P450 reductase transcript levels found in the CBS*-CYP51*^+/−^strain after 24 h of cultivation (see below) that can sustain cytochrome P450 activity during ergosterol biosynthesis.

Similar to our findings, several studies have observed increased carotenoid production in *X. dendrorhous* when ergosterol levels are reduced. For example, when *Phaffia rhodozyma* (the anamorphic state of *X. dendrorhous*) was cultured in the presence of the antifungal ergosterol biosynthesis inhibitor fluconazole, enhanced astaxanthin production was observed [42]. One possible explanation is that ergosterol down-regulates its own synthesis via a negative-feedback mechanism. Therefore, a reduced ergosterol content favors the availability of mevalonate pathway products, which are common to ergosterol and carotenoid syntheses. This reasoning is supported by a previous study in which *X. dendrorhous* mutants that are unable to synthesize ergosterol due to the *cyp61* mutation had increased carotenoid content compared to their parental wild type strains. Moreover, transcript levels of at least one gene of the mevalonate pathway (*HMGR* that encodes the hydroxymethylglutaryl CoA-reductase) were significantly increased [20]. In this sense, the supplementation of mevalonate in *P. rhodozyma* cultures resulted in an increase in carotenoid production [43]. Furthermore, deletion of the gene encoding squalene synthase (*ERG9*, the first step of sterol biosynthesis *per se*) in combination with the overexpression of the catalytic domain of the hydroxymethylglutaryl CoA-reductase in a recombinant *Candida utilis* strain caused an increase in lycopene production [44].

**Figure 4:**
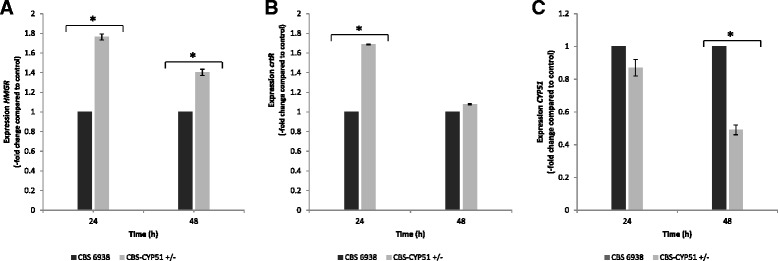
RT-qPCR analysis of the *HMGR, crtR* and *CYP51* genes in the wild-type and CBS*-CYP51* ^+/−^ strains. The *HMGR* **(A)**, *crtR* **(B)** and *CYP51* **(C)** transcript levels were determined by RT-qPCR after 24 and 48 h of cultivation of the wild-type and the CBS*-CYP51* ^+/−^strains. Each transcript level was normalized with respect to the transcript level of the actin gene and then with respect to the wild-type strain: CBS 6938 (=1, black bars) and CBS-CYP51^+/−^ (gray bars). Values are the mean ± standard error of three independent experiments (* p ≤ 0.05; Student’s *t* test).

Based on this reasoning, we evaluated whether *HMGR* transcript levels were enhanced in strain CBS-CYP51^+/−^. To accomplish this, total RNA was extracted from the parental and CBS-CYP51^+/−^ strains after 24 and 48 h of cultivation in YM media with constant agitation at 22°C, and the transcript levels were analyzed by RT-qPCR. The results revealed that the *HMGR* transcript level was indeed higher in the mutant strain compared to the wild-type strain, indicating that the elimination of one allele of the *CYP51* gene of *X. dendrorhous* affected the expression of *HMGR* (Figure 4A). The *HMGR* gene controls one of the key regulatory steps in the mevalonate pathway, and, in turn, carotenoid and ergosterol biosynthesis through substrate availability [45]; therefore, an increase in the *HMGR* transcript level could partly explain the increased carotenoid content in the mutant strain CBS-*CYP51*^+/−^. Similar to our findings, there was an increase in carotenoid content that was also related to a higher *HMGR* transcript levels following the treatment of the fungus *Blaskelea trispora* with ketoconazole, a specific inhibitor of CYP51 [46].

Because cytochrome P450 enzymes are involved in both astaxanthin and ergosterol biosynthesis, we also evaluated the *crtR* transcript levels [12]. *crtR* encodes for a cytochrome P450 reductase, which is the main electron donor in these systems. Interestingly, the *crtR* transcript level was also higher in CBS-CYP51^+/−^ after 24 h of cultivation; however, this difference was not statistically significant after 48 h of growth (Figure 4B). Although several different genes may encode cytochrome P450 enzymes in an organism, usually there is only one gene encoding the enzyme cytochrome P450 reductase. Consequently, a complex mechanism of regulation of the expression of *crtR* is required to set the levels of cytochrome P450 reductase activity in relation to the various cytochrome P450 proteins in this organism. To date, three cytochrome P450-encoding genes have been described in *X. dendrorhous*: *crtS* [10], *CYP61* [20] and *CYP51* [this current work]). Thus, the cytochrome P450 reductase would act as a cytochrome P450 redox partner at least in the carotenoid and ergosterol synthesis pathways. Finally, we also analyzed whether the elimination of one *CYP51* allele indeed affected its own transcript levels. After 24 h of cultivation, no significant differences were observed between the mutant and the parental strain; however, after 48 h of cultivation, differences could be appreciated (Figure 4C), confirming that the *X. dendrorhous* mutant generated in this work indeed affected *CYP51* transcript levels.

## Conclusions

In this work, the *CYP51* gene from *X. dendrorhous* was identified and its function was characterized. This gene encodes a putative protein that contains all of the cytochrome P450 conserved motifs and is consistent with CYP51 protein family characteristics. The heterologous complementation analysis in *S. cerevisiae* strongly suggests that this gene in fact encodes a functional sterol C14-demethylase. Moreover, a *CYP51* mutation in *X. dendrorhous* affected sterol and carotenoid production, supporting the finding that reduced sterol production is related to higher carotenoid production.

## Methods

### Biological material and microorganism culture conditions

Restriction enzymes, the Klenow polymerase and the M-MLV reverse transcriptase were purchased from Promega, and the *Pfu* DNA polymerase was purchased from Invitrogen. The plasmids and microbial strains that were used and constructed for this work are included in Table 3. The wild type UCD 67–385 (ATCC 24230) *X. dendrorhous* strain was used for genome and transcriptome sequencing and for genomic and cDNA *CYP51* gene amplification and isolation. For the *CYP51* gene mutation, the wild type CBS 6938 (ATCC 96594) *X. dendrorhous* strain was used. *Escherichia coli* DH-5α was used as a host for plasmid propagation. *Saccharomyces cerevisiae* Meyen ex E.C Hansen YHR007C BY4743 (ATCC 26604) was used for *CYP51* heterologous complementation analysis because it is a diploid and heterozygous mutant for the *ERG11* gene (*erg11*^(+/−)^), with one copy of *ERG11* replaced by a geneticin resistance cassette. Most plasmid constructs derived from pBluescript SK- [47]. Plasmid YEpNP was modified from YEpACT4 [48] and used for *S. cerevisiae* heterologous complementation.

**Table 3:** **Plasmids and strains used and constructed in this study**

	**Relevant features or strain genotype**	**Source or Reference**
**Plasmids:**		
pBluescript SK- (pBS)	ColE1 ori; AmpR; cloning vector with blue-white selection.	Invitrogen
pMN-*hph*	pBS containing at the *Eco*RV site a cassette of 1.8 kb bearing the *E. coli*-Hygromycin B resistance (*hph*) gene under EF-1 α promoter and *GPD* transcription terminator of *X. dendrorhous*.	[56]
pBS-g*CYP51*	pBS bearing the genomic version of the *CYP51* gene from *X. dendrorhous* (4,318 bp).	This work
pBS-c*CYP51*	pBS bearing the ORF of the *CYP51* gene from *X. dendrorhous* (1,653 bp).	This work
pBS-*cyp51*/*hph*	pBS bearing the hygromycin B resistance cassette flanked by approximately 700 bp upstream and 600 pb downstream of the *X. dendrorhous CYP51 locus*.	This work
YEp-Act4	pBR322 and 2 micron ori, Amp^R^, *LEU2* and containing the *ACT* promoter to regulate gene expression.	[48]
YEpNP	YEp-Act4 bearing the *S. cerevisiae TDH3* terminator.	This work
YEpNP-c*CYP51*	YEpNP bearing the *X. dendrorhous CYP51* cDNA (1,653 bp) under the regulation of the *ACT* promoter and the *TDH3* terminator.	This work
YEpNP-g*ERG11*	YEpNP bearing the *S. cerevisiae ERG11* gene (1,593 bp) under the regulation of the *ACT* promoter and the *TDH3* terminator.	This work
**Strains:**		
*E. coli*:		
DH-5α	F- φ80d lacZΔM15Δ (lacZY-argF) U169 deoR recA1 endA1 hsdR17(rk- mk+) phoA supE44 l- thi-1 gyrA96 relA1	[47]
*S. cerevisiae:*		
S288c	MATα, SUC2, gal2, mal, mel, flo1, flo8-1, hap1, ho, bio1, bio6.	[64]
Meyen ex E.C Hansen YHR007C BY4743 (Sc-*erg*11^+/−^)	MATa/MATα, his3Δ1/ his3Δ1, leu2Δ0/leu2Δ0, lys2Δ0/+, met15Δ0/+, ura3Δ0/ura3Δ0, ΔERG11:KanMx. ATCC 26604.	ATCC 26604
Sc-c*CYP51*Xd	Diploid transformant derived from Sc-*erg11* ^+/−^ containing the expression vector YEpNP-c*CYP51*.	This work
Sc-g*ERG11*Sc	Diploid transformant derived from Sc-*erg11* ^+/−^ containing the expression vector YEpNP-g*ERG11*.	This work
Sc-h*CYP51*	Sporulation product derived from Sc-*erg11* ^+/−^ containing the expression vector YEpNP- c*CYP51*.	This work
Sc-h*ERG11*	Sporulation product derived from Sc-*erg11* ^+/− ^containing the expression vector YEpNP-g*ERG11*.	This work
*X. dendrorhous:*		
UCD 67-385	ATCC 24230, wild-type.	ATCC 24230
CBS 6938	ATCC 96594, wild-type.	ATCC 96594
CBS*-CYP51* ^+/−^	(CBS-*CYP51*/*cyp51* ^*hph*^). Transformant derived from CBS 6938 containing an allele of the *CYP51 locus* replaced by a hygromycin B resistance module.	This work

*X. dendrorhous* was cultured in YM rich medium (1% glucose, 0.3% yeast extract, 0.3% malt extract and 0.5% peptone) with constant agitation at 22°C. For transformant selection, the yeast was grown on 1.5% agar YM plates supplemented with 10 μg/ml hygromycin B (US Biological).

*E. coli* was cultured at 37°C with constant agitation in Luria-Bertani (LB, 1% tryptone, 0.5% yeast extract, and 0.5% NaCl) medium and on 1.5% agar LB plates supplemented with 100 μg/ml ampicillin for plasmid selection and 20 μg/ml of X-gal (5-bromo-4chloro-3-indolyl-β-D-galactopyranoside) for recombinant clone selection by blue-white screening [47]. For selection, recombinant clones bearing the constructed plasmids were analyzed by direct colony PCR with an adequate set of primers.

*S. cerevisiae* strains were cultured at 30°C in YM or YEP (YM: 0.3% yeast extract, 0.3% malt extract and 0.5% peptone; YEP: 2% glucose, 1% yeast extract, and 2% peptone) rich media. The strain Meyen ex E.C Hansen YHR007C BY4743 was cultured at 22°C in SD medium (0.67% yeast nitrogen base without amino acids and 2% glucose) supplemented with metabolites to sustain the strain auxotrophy (0.002% uracil, 1% histidine and 1% leucine) and 200 μg/ml of geneticin. Transformants derived from this strain were cultured in SD medium supplemented with uracil, histidine and geneticin, because the vector YEpNP complements the parental strain’s leucine auxotrophy.

### Nucleic acid extraction, DNA amplification and sequence analysis

*X. dendrorhous* DNA extraction was performed from protoplasts according to [49], and total RNA extraction was performed according to a modified protocol of Chomczynski and Sacchi [50] for *X. dendrorhous* [38]. Total RNA was quantified spectrophotometrically at 260 nm according to [47] in a V-630 UV–vis Spectrophotometer (JASCO). *S. cerevisiae* genomic DNA was obtained by mechanical cell disruption using 0.5 mm glass beads (BioSpec) and shaking in a mini bead beater-16 (BioSpec). Plasmid DNA was obtained from *E. coli* strains using the AxyPrep Plasmid Miniprep kit (Axygene).

Oligonucleotides designed and used in this work were synthesized by Integrated DNA Technologies and are listed in Additional file 2: Table S1. PCR reactions were performed in a final volume of 25 μl with 2 U of *Taq* DNA polymerase, 2.5 μl of 10X *Taq* buffer, 0.5 μl of 10 mM dNTPs, 1 μl of 50 mM MgCl_2_, 1 μl of each primer (25 μM) and 10 to 20 ng of template DNA in an Applied Biosystems 2720 thermal cycler. The general amplification protocol was as follows: initial denaturation at 95°C for 3 min; 35 cycles of denaturation at 94°C for 30 s, annealing at 55°C for 30 s, and synthesis at 72°C for 3 min; and a final extension step at 72°C for 10 min. Samples were stored at 4°C until analyzed by 0.8% agarose gel electrophoresis in TAE buffer stained with 0.5 μg/ml ethidium bromide [47]. DNA for sequencing or plasmid construction was purified from gels by the glass milk method [51].

DNA sequencing was performed in an ABI 3100 Avant genetic analyzer using the BigDye terminator v3.1 kit (Applied Biosystems) and analyzed with Vector NTI Suite 10 (Informax), CLUSTAL W 1.8 and programs available at the NCBI web site. Protein sequence analyses were performed with programs available at www.ch.embnet.org/software/TMPRED_form.html [52], www.ebi.ac.uk/InterProScan/ [53] and www.cyped.uni-stuttgart.de/ [25].

Protein modeling was performed using programs available at www.swissmodel.expasy.org/ [54], http://autodock.scripps.edu/wiki/AutoDock4 [31] and www.ks.uiuc.edu/Research/vmd/ [55].

### Isolation of the *X. dendrorhous CYP51* gene and plasmid construction

To identify the *CYP51* gene from *X. dendrorhous,* BLAST analyses using homologous *CYP51* nucleotide and amino acid sequences were performed with the yeast genomic and transcriptomic databases available in our laboratory [20]. Specific primers were designed from the identified sequences (Additional file 2: Table S1).

For the *S. cerevisiae* heterologous complementation experiments, the *CYP51* cDNA from *X. dendrorhous* and the *ERG11* gene from *S. cerevisiae* (homologous to *CYP51*) were ligated into the yeast expression plasmid YEpNP (Table 3). First, plasmid pBS-cCyp51, which contained the cDNA of the *CYP51* gene, was constructed by inserting a 1,653 bp RT-PCR product into the *Eco*RV site of plasmid pBluescript SK-, which was obtained using the primer set cCYP51.F + cCYP51.R (Additional file 2: Table S1). Plasmid pBS-cyp51/Hyg was used to eliminate an allele of the *CYP51* gene in *X. dendrorhous*. This plasmid was obtained by sequentially joining three DNA fragments: i) 700 bp (amplified with primers CYP51_del.Fw + CYP51_del-HpaI.Rv) upstream of the *CYP51* gene encoding sequence; ii) the hygromycin B resistance module [56] (amplified with primers H.F and H.R); and iii) 600 bp (amplified with primers CYP51_del-HpaI.Fw + CYP51_del.Rv) downstream of the *CYP51* gene encoding sequence. These sequences were inserted into the *Eco*RV site of plasmid pBluescript SK-. For *X. dendrorhous* transformation, the insert of plasmid pBS-cyp51/Hyg was released using the *Not*I and *Xho*I endonucleases.

### Yeast transformation

*S. cerevisiae* electrocompetent cells were prepared from 60 ml of exponentially growing cultures in YEP medium at 22°C with constant agitation. The cells were washed three times with chilled sterile distilled water, one time with sorbitol 1 M and finally suspended in 0.2 ml of 1 M sorbitol. The cells were kept at 4°C, and 40 μl aliquots were used for electroporation with a BioRad gene pulser × cell with PC and CE modules under the following conditions: 1.5 kV, 25 μF and 200 Ω. *X. dendrorhous* transformation was also performed by electroporation according to [57] and [58] using 1 to 5 μg of linear donor DNA. Electrocompetent cells were prepared from exponential cultures, grown in YM medium and electroporated using the following program: 125 mF, 600 Ω, 0.45 kV.

### *S. cerevisiae* heterologous complementation assay

The *erg11*^(+/−)^*S. cerevisiae* strain, which is diploid and heterozygous for the *ERG11* gene, was used for the gene complementation assays. The *S. cerevisiae ERG11* gene and the *X. dendrorhous CYP51* gene were ligated into plasmid YEpNP and used to independently transform the original diploid yeast strain. Transformants were selected in SD agar plates supplemented with 200 μg/ml geneticin, 0.002% uracil and 1% histidine according to the parental strain requirements. Transformant strains were randomly chosen and incubated in pre-sporulation medium plates (0.8% yeast extract, 0.3% peptone, 10% glucose and 2% agar), incubated at 22°C for 1 to 2 days and then transferred to minimal sporulation medium plates (1% potassium acetate and 2% agar) for incubation at 22°C for 3 to 6 days. Supplements according to the parental strain genotype were added to the sporulation media. Asci formation was confirmed by optical microscopy. To enrich the asci fraction, cells were collected from the sporulation plates, treated with diethyl ether for 20 min at 22°C [34] and the ascospores were released by treatment with zimoliase-120 T (US Biological) at 30°C for 2 h. Afterwards, the mixture was shaken in a mini bead beater-16 (BioSpec) with 0.5 mm glass beads (BioSpec) for 2 min [59]. Finally, the cells were spread on SD agar plates supplemented with histidine, uracil, lysine, methionine and geneticin to support all possible combinations of auxotrophies in the resulting haploid cells.

To select the desired haploid cells to study gene complementation, the obtained colonies were streaked in replicates on SD agar plates supplemented with geneticin and: i) uracil, histidine and lysine, ii) uracil, histidine and methionine and iii) uracil, histidine, methionine and lysine. Colonies that developed on the plates supplemented with the four metabolites but not when lysine or methionine were omitted were chosen for further analyses.

### Sterol and carotenoid extraction and analysis

Sterols were extracted from both the *S. cerevisiae* and *X. dendrorhous* strains, while carotenoids were extracted only from *X. dendrorhous*. Metabolites were spectrophotometrically quantified and normalized to the dry weight of the yeast.

Sterols were extracted according to the protocol of [60] by mixing cell pellets with 4 g of KOH and 16 ml of 60% (v/v) ethanol/water and incubating at 80 ± 2°C for 2 h. Then, non-saponificable sterols were extracted with 10 ml of petroleum ether and quantified spectrophotometrically at 280 nm. Carotenoids were extracted from cellular pellets by the acetone method [61] and quantified spectrophotometrically at 465 nm. Sterol and carotenoid compositions were analyzed by RP-HPLC with an RP-18 Lichrocart125-4 (Merck) column using methanol: water (97: 3, v/v) or acetonitrile: methanol: isopropanol (85: 10: 5, v/v) as the mobile phase with a 1 ml/min flux under isocratic conditions to separate the sterols and carotenoids, respectively. The elution spectra were recovered using a diode array detector, and the metabolites were identified according to their spectra and retention time in comparison to standards.

### Single-strand DNA synthesis and RT-qPCR

Single-stranded DNA was synthesized according to the M-MLV reverse transcriptase (Invitrogen) manufacturer’s instructions using 5 μg of total RNA in a final volume of 20 μl. Relative gene expression levels were obtained in an Mx3000P quantitative PCR system (Stratagene) using 1 μl of the reverse transcription reaction, 0.25 μM of each primer and 10 μl of the SensiMix SYBR Green I (Quantace) kit in a final volume of 20 μl. The Ct values obtained were normalized to the corresponding value for the beta-actin encoding gene [Genbank: X89898.1] [62], and later expressed as a function of the control conditions using the ∆∆Ct algorithm [63].

## Additional files

Additional file 1: Figure S1. Growth curves of yeast strains used in this study. (A) *S. cerevisiae* strains Sc-*erg*11^+/−^, Sc-h*ERG11* and Sc-h*CYP51* were cultured in YM media at 22°C with constant agitation. (B) Growth curve of the *X. dendrorhous* CBS 6938 and CBS*-CYP51* ^+/−^ strains cultured in YM media at 22°C with constant agitation. Values are the mean ± standard error of three independent cultures. Additional file 2: Table S1. Primers designed and used in this study.
