# Supplementary figures and images for: Identification and functional characterization of the CYP51 gene from the yeast Xanthophyllomyces dendrorhous that is involved in ergosterol biosynthesis
Source: BMC Microbiol. 2015 Apr 25;15:89. doi: 10.1186/s12866-015-0428-2 (PMC4415319; doi:10.1186/s12866-015-0428-2)

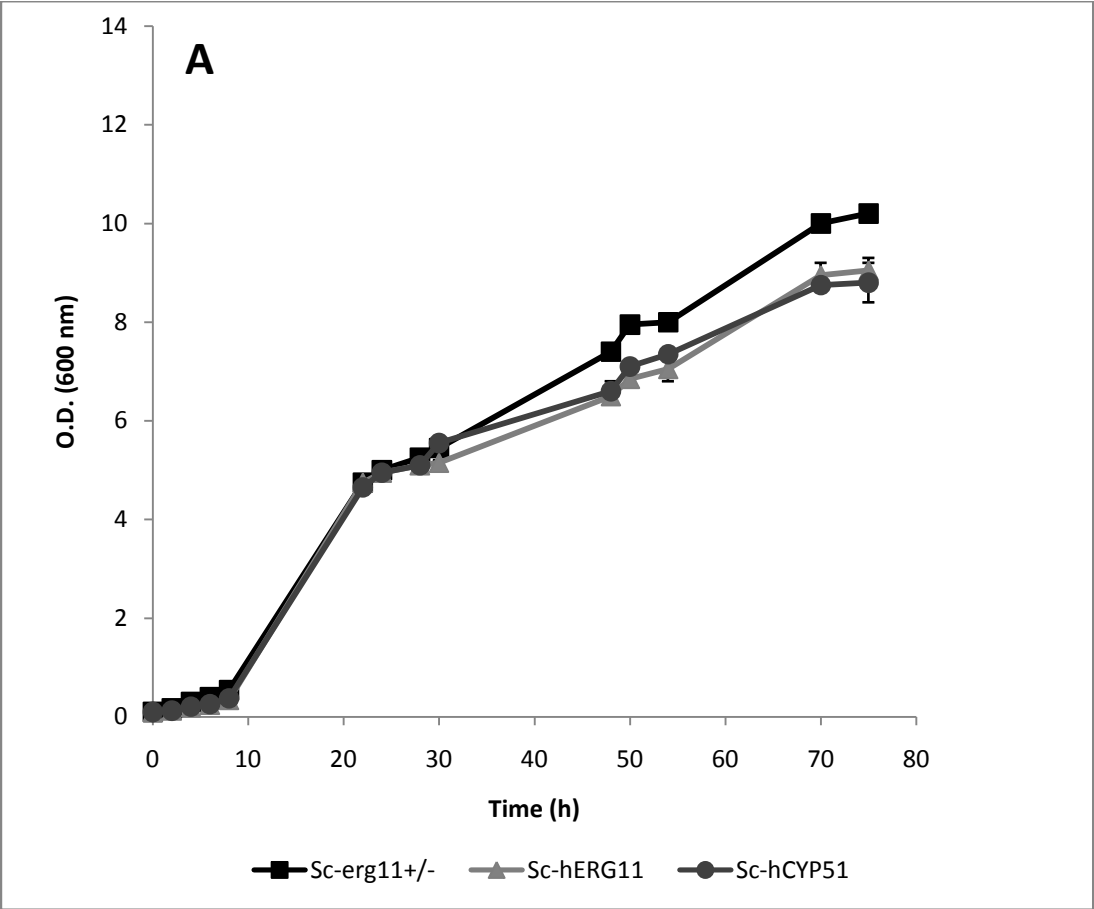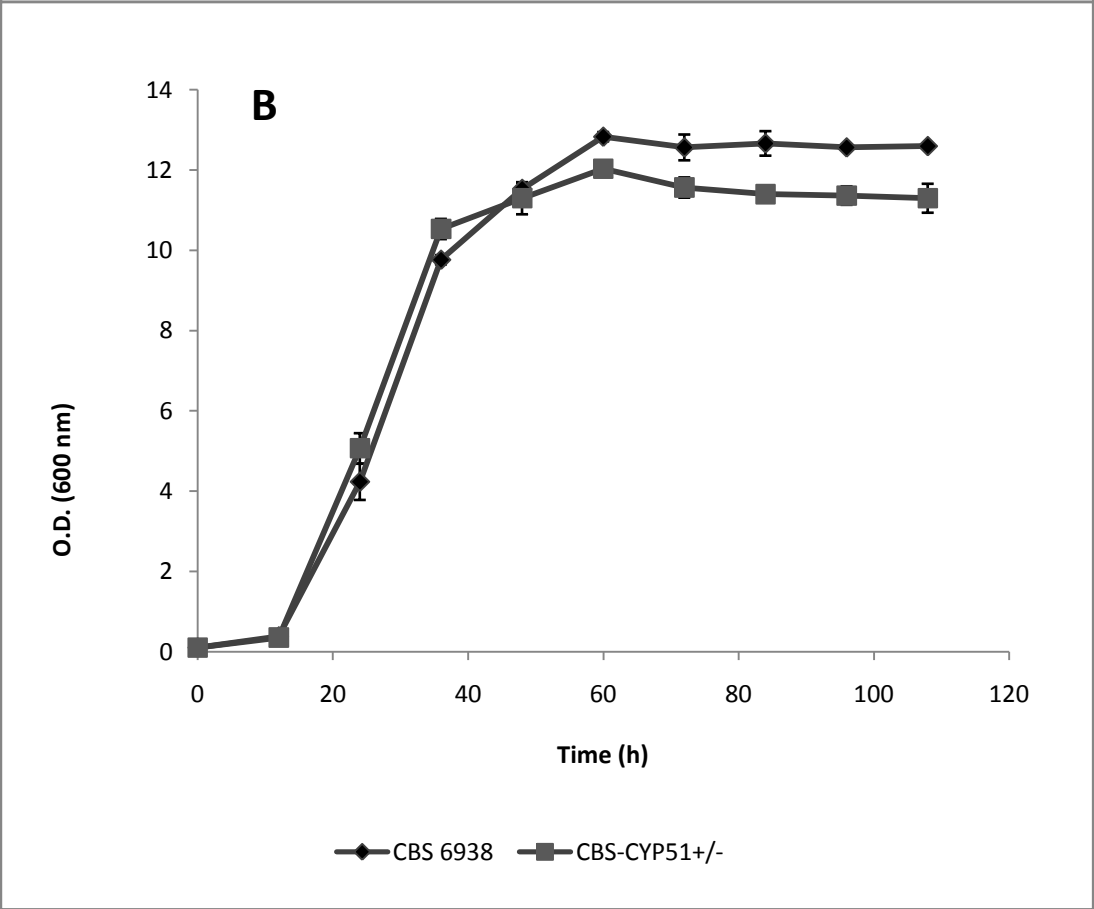

Supplement: Additional file 1: Figure S1. — Growth curves of yeast strains used in this study. (A) S. cerevisiae strains Sc-erg11+/−, Sc-hERG11 and Sc-hCYP51 were cultured in YM media at 22°C with constant agitation. (B) Growth curve of the X. dendrorhous CBS 6938 and CBS-CYP51 +/− strains cultured in YM media at 22°C with constant agitation. Values are the mean ± standard error of three independent cultures. [file 12866_2015_428_MOESM1_ESM.pdf]
