# Supplementary material for: Identification and functional characterization of the CYP51 gene from the yeast Xanthophyllomyces dendrorhous that is involved in ergosterol biosynthesis
Source: BMC Microbiol. 2015 Apr 25;15:89. doi: 10.1186/s12866-015-0428-2 (PMC4415319; doi:10.1186/s12866-015-0428-2)
Supplement: Additional file 2: Table S1. — Primers designed and used in this study. [file 12866_2015_428_MOESM2_ESM.docx]

### Additional file 2 – Table S1: Primers designed and used in this work.

| **Primer** | **Sequence (5' to 3')** | **Target** |
| --- | --- | --- |
| M13.Fw.FP | tgtaaaacgacggccagt | M13 Region from vector YEpNP. |
| M13.Rv.PUC-40 | ggaaacagctatgaccatg | M13 Region from vector YEpNP. |
| KanMX4.F2 | ttgtatgggaagcccgatg | G-418 resistance cassette. |
| KanMX4.R2 | gatcctggtatcggtctgc | G-418 resistance cassette. |
| H.F | atgaaaaagcctgaactcacc | *hph* gene (hygromycin B resistance cassette)*.* |
| H.R | ctattcctttgccctcggac | *hph* gene (hygromycin B resistance cassette)*.* |
| TEF_Xd.F | gggctcatcagccgacagttca | *X. dendrorhous* *TEF* promoter (hygromycin B resistance cassette)*.* |
| TEF_Xd_antisense.R | tcctaattcttgtcgacaacg | *X. dendrorhous* *TEF* promoter (hygromycin B resistance cassette)*.* |
| GPD_Xd_sense.F | ggacaaggcaagaagtgagca | *X. dendrorhous* *gpd* terminator (hygromycin B resistance cassette)*.* |
| GPD_Xd.R | atgagagatgacggagatgat | *X. dendrorhous* *gpd* terminator (hygromycin B resistance cassette)*.* |
| gERG11.F | atgtctgctaccaagtcaatcg | *S. cerevisiae ERG11* gene*.* |
| gERG11.R | ttagatcttttgttctggatttc | *S. cerevisiae ERG11* gene*.* |
| CYP51ScExt.F | ttgccctccatgtgtattc | Upstream region of the *S. cerevisiae ERG11 locus.* |
| CYP51ScExt.R | aatccagtaggcatgttgc | Downstream region of the *S. cerevisiae ERG11 locus.* |
| CYP51ATG.F | atgtcgtcgtcccaatcgatc | cDNA version of the *X. dendrorhous CYP51* gene*.* |
| CYP51TAG.R | ctacgcggcagcctttcg | cDNA version of the *X. dendrorhous CYP51* gene*.* |
| CYP51_Rb | ctggtagggagattcagtcc | cDNA version of the *X. dendrorhous CYP51* gene*.* |
| cCYP51.F | cctttgctcgtcgttttctc | cDNA version of the *X. dendrorhous CYP51* gene*.* |
| cCYP51.R | tcgcccaaatttgaagagac | cDNA version of the *X. dendrorhous CYP51* gene*.* |
| Pre_CYP51_del.F | ccaagttcctgtgtgctgagt | Upstream region of the *X. dendrorhous CYP51* gene*.* |
| Post_CYP51_del.R | tgcctggataagaaatgaggg | Downstream region of the *X. dendrorhous CYP51* gene*.* |
| Cyp51_del.Fw | catgaaagtggggtcacacga | To construct pBS-*cyp51*/*hph*: 0.7 kb upstream the *X. dendrorhous CYP51* gene start codon. |
| Cyp51_del-HpaI.Rv | aagcgtctgat**gttaac***aatcagaaagatcgagtcta* | To construct pBS-*cyp51*/*hph*: 11 bases of the *X. dendrorhous CYP51* gene terminator region (underlined), *Hpa*I restriction site (bold) and 20 bases of the *X. dendrorhous CYP51* gene promoter region (italics)*.* |
| Cyp51_del.Rv | aacgagatgatacgtaccgac | To construct pBS-*cyp51*/*hph*: 0.6 kb downstream the *X. dendrorhous CYP51* gene stop codon. |
| Cyp51_del-HpaI.Fw | gatctttctgatt**gttaac***atcag acgcttttccttga* | To construct pBS-*cyp51*/*hph*: 13 bases of the *X. dendrorhous CYP51* gene promoter region (underlined), *Hpa*I restriction site (bold) and 19 bases of the *X. dendrorhous CYP51* gene terminator region (italics)*.* |
| CYP51up.F | cgttcttgtggagtctgtgc | *X. dendrorhous CYP51* gene*.* |
| CYP51dw.R | tcggaccttgacatagactgc | *X. dendrorhous CYP51* gene*.* |
| RTCYP51.F | cagctcgctcagttgattcctaga | cDNA version of the *X. dendrorhous CYP51* gene (RT-qPCR analysis). |
| RTCYP51.R | atgtgaacagatcgccgtgctt | cDNA version of the *X. dendrorhous CYP51* gene (RT-qPCR analysis). |
| HMGR-real-Fw1 | ccaaacgatggttggattcggt | cDNA version of the *X. dendrorhous HMGR* gene*.* (RT-qPCR analysis). |
| HMGR-real-Rv1 | tccgttcctttaccagccatgt | cDNA version of the *X. dendrorhous HMGR* gene (RT-qPCR analysis). |
| mcrtRR-RT-F | ctgggaaacaagacctacga | cDNA version of the *X. dendrorhous crtR* gene (RT-qPCR analysis) |
| mcrtRR-RT-R | ggaacctcggttacgacaaa | cDNA version of the *X. dendrorhous crtR* gene (RT-qPCR analysis). |
| mActF-RT | ccgccctcgtgattgataac | cDNA version of the *X. dendrorhous actin* gene (RT-qPCR analysis) |
| mActR-RT | tgaccaaggtaggagtcctt | cDNA version of the *X. dendrorhous actin* gene (RT-qPCR analysis). |
